# Circulating Hsp90 Isoform Levels in Overweight and Obese Children and the Relation to Nonalcoholic Fatty Liver Disease: Results from a Cross-Sectional Study

**DOI:** 10.1155/2019/9560247

**Published:** 2019-12-03

**Authors:** Anca Bălănescu, Iustina Stan, Ioana Codreanu, Valentina Comănici, Eugenia Bălănescu, Paul Bălănescu

**Affiliations:** ^1^Pediatrics Chair, University of Medicine and Pharmacy “Carol Davila”, 37 Dionisie Lupu Street, Bucharest, Romania; ^2^National Institute for Mother and Child Health “Alessandrescu-Rusescu”, 120 Lacul Tei Avenue, Bucharest, Romania; ^3^CDPC Clinical Immunology Department, Colentina Clinical Hospital, 19-21 Stefan cel Mare Street, Bucharest, Romania; ^4^Clinical Research Unit RECIF (Reseau d'Epidemiologie Clinique International Francophone), 19-21 Stefan cel Mare Street, Bucharest, Romania; ^5^Internal Medicine Chair, University of Medicine and Pharmacy “Carol Davila”, 37 Dionisie Lupu Street, Bucharest, Romania

## Abstract

**Background:**

Obesity prevalence is increasing in children. It is associated with various comorbidities including nonalcoholic fatty liver disease (NAFLD). Hsp90 isoforms were identified in previous proteomic studies as potential biomarkers for NAFLD. The aim of the study was to analyze circulating levels of Hsp90*α* and Hsp90*β* in overweight and obese children. In addition, Hsp90*α* and Hsp90*β* were evaluated as biomarkers for NAFLD in overweight and obese children.

**Methods:**

68 overweight and obese children and ten age- and gender-matched controls were recruited. Hsp90*α* and Hsp90*β* levels were analyzed from serum in both controls and overweight and obese children by ELISA.

**Results:**

Serum Hsp90*β* and total Hsp90 levels were statistically significantly higher in overweight and obese children compared to controls. On the contrary, there was no difference in Hsp90*α* levels between overweight and obese children and healthy controls. Hsp90 isoforms had different expression in NAFLD patients. Hsp90*β* levels were higher in overweight and obese NAFLD patients while Hsp90*α* levels were lower. Hsp90*α* to Hsp90*β* ratio had better accuracy for NAFLD diagnosis in obese and overweight patients compared to individual biomarkers.

**Conclusion:**

Hsp90 isoforms were confirmed on an independent cohort as biomarkers for NAFLD in overweight and obese children. In these patients, it seems to be more useful to separately analyze Hsp90 isoforms rather than total Hsp90 as the isoforms have greater discriminative capacity.

## 1. Introduction

Obesity is a worldwide problem and unfortunately its prevalence in children is increasing [1]. Obesity is associated with various comorbidities including insulin resistance, hypertension, and nonalcoholic fatty liver disease (NAFLD) [2]. Also, obesity is a risk factor for NAFLD development. NAFLD encompasses various hepatic lesions, from simple to progressive steatosis and inflammation, fibrosis, cirrhosis, and eventually hepatocellular carcinoma [3]. Its prevalence is increasing in childhood, and research efforts are made in order to find early-onset predictors for NAFLD in obese children [4]. Biomarkers for NAFLD are of paramount importance for early diagnosis and implementation of preventive strategies in order to decrease the burdens associated in adulthood.

Heat shock proteins (Hsp, stress proteins) are a heterogeneous class of proteins with essential roles like protein folding, protein sorting, and presentation of antigen [5]. Hsp90 is a ubiquitous molecular chaperone implied in cell survival, signal transduction, and cellular response against various stressors [6]. It is present in the cytoplasm of the cell under several isoforms, but the most important ones are Hsp90*α* (major and inducible form) and Hsp90*β* (minor and constitutive form) [7].

Previous proteomic studies [8] indicated the Hsp90 family having differential expression in adult obese NAFLD patients versus healthy control individuals, proposing them as potential biomarkers for NAFLD. However, there is no previous study addressing the Hsp90 family in a pediatric obese group. Specifically, these biomarkers should be confirmed in an independent cohort using specific detection methods based on antibody detection. The aim of the study was to examine circulating levels of Hsp90*α* and Hsp90*β* in overweight and obese children. In addition, Hsp90*α* and Hsp90*β* were evaluated as biomarkers for NAFLD in overweight and obese children.

## 2. Materials and Methods

### 2.1. Patients

Two separate children groups were recruited. One group consisted of 68 overweight and obese children from MRPONy cohort (“Metabolic and cardiovascular Risk factors in a Pediatric Obese population with or without NAFLD”) [9], and the second group consisted of ten gender- and age-matched healthy controls. From January 2017 until April 2018, patients and controls were consecutively recruited from patients that referred to the Pediatrics Department from INSMC Alessandrescu-Rusescu, Bucharest, Romania. All patients and/or the legal guardian gave an informed consent. The study was approved by the local ethics committee of the National Institute for Mother and Child Health (INSMC) “Alessandrescu-Rusescu.” Eligible patients had to have age between 3 and 18 years and to be overweight (body mass index (BMI) greater than the 85th percentile for gender and age) or obese (BMI above the 95^th^ percentile for gender and age). Patients that had other causes of hepatocytolysis, genetic traits, endocrine disorders, and acute inflammatory diseases were excluded. As previously presented, all patients underwent standardized clinical and biochemical profiling [9]. Briefly, anthropometric evaluation was recorded in all patients. Every patient underwent a standardized blood pressure measurement according to “WHO STEPS surveillance manual: the WHO STEPwise approach to chronic disease risk factor surveillance/Noncommunicable Diseases and Mental Health, World Health Organization” [10]. Liver steatosis was assessed by abdominal ultrasound using the Toshiba Aplio 300 Ultrasound Machine. All children were evaluated by the same ecographist, and all measurements were carried out using the same device. Fatty liver disease (NAFLD) was defined when ultrasound changes were found and/or the patient had elevated alanine transaminase (ALAT) levels. Current literature specifies relevant elevated ALAT level as being more than twice the normal upper limit [11]. Ultrasound changes that were taken into consideration were summarized by Joy et al. [12] and consisted of increased hepatic echogenicity compared with spleen and renal cortex echogenicity, increased attenuation of the ultrasound beam (posterior darkness) passing through the liver with loss of definition of the diaphragm and loss of intrahepatic architectural detail (portal veins not being well defined).

Blood samples were taken from each participant, and serum was stored at -80°C for further analysis of the biomarkers of interest. In order to test for insulin resistance, HOMA-IR index (homeostasis model assessment) was calculated for each individual from fasting samples [13]. Insulin was dosed using an “in vitro” diagnosis ELISA kit provided by Mercodia Ultrasensitive Insulin Assay. Samples were analyzed as indicated by the kit manufacturer. Optic absorbance was analyzed using ELISA plate reader from Bio-Rad. Insulin resistance was considered if the HOMA-IR values were higher than 2.5 for prepubertal children and above 4 for pubertal children (according to Calcaterra et al. [14]). ASAT, ALAT, alkaline phosphatase, and uric acid were spectrophotometrically analyzed using Dimension RXL Max chemistry system.

### 2.2. Serum Hsp90 Analysis

Serum stored at -80°C was further analyzed for circulating Hsp90*α* and Hsp90*β* expression using commercial ELISA kits. Hsp90*α* was analyzed from serum samples in duplicate using ELISA kit from Novus Biologicals (limit of detection 0.03 ng/mL, intra-assay coefficient variation 8.6%, interassay coefficient variation 10%). The analysis was performed according to manufacturer instructions. Hsp90*β* was analyzed from serum samples in duplicate with ELISA kit from CUSABIO (limit of detection 0.1 ng/mL, intra-assay coefficient variation 8%, interassay coefficient of variation 10%). There were no reported cross-reactivities for both ELISA kits used. The analysis was performed according to manufacturer instructions. Incubations, washing steps, and optic absorbance were performed using the ELISA system from Bio-Rad. Mean optical absorbance from duplicates was considered and transformed into concentration after having been compared to a standard 4PL curve, as indicated by the manufacturers. Serum Hsp90*α* and Hsp90*β* concentrations were expressed in the same manner, as ng/mL.

Total Hsp90 serum levels were estimated by adding Hsp90*α* with Hsp90*β* values.

### 2.3. Statistical Approach

Continuous variables were summarized by mean and standard deviation if they had normal distribution or as median and minimum-maximum value if the distribution was not normal. Categorical variables were summarized as value and percent. Parametric tests were used to assess differences between continuous variables if distribution was normal, or nonparametric tests were used if the distribution was not normal. Chi-square tests were used to analyze differences between categorical variables. SPSS version 16 (Chicago, USA) was used for statistical analysis. Power analysis was performed using ClinCalc sample size calculator (https://clincalc.com/stats/samplesize.aspx). In order to detect a difference of 5 ng/mL for Hsp90*α* and 1 ng/mL for Hsp90*β* levels with an allocation rate of 1/3, for an *α* error probability at 0.05 and for a study power of 0.80, a minimum of ten controls and 30 obese patients would be needed.

## 3. Results

The overweight and obese group consisted of 68 children: 35 boys (51.5%) and 33 girls (48.5%). Their median age was 10 years (3 years-17 years). 25 patients (36.8%) had metabolic syndrome as defined by IDF consensus criteria [15]. 26 patients (38.2%) were considered to have NAFLD with 7 patients out of these 26 having severe hepatocytolysis and were considered with NASH (nonalcoholic steatohepatitis). Median HOMA-IR index was 2.20 (0.34-13.57). Patient characteristics are summarized in Table 1. The control group consisted of ten gender- and age-matched healthy children, 3 girls (30%) and 7 boys (70%) (*p* = 0.33 compared to the overweight and obese group, exact Fisher test) with a median age of 9 years (4 years-16 years, *p* = 0.76, compared to the overweight and obese group, Mann-Whitney *U* test).

Serum Hsp90*β* and total Hsp90 levels were statistically significantly higher in overweight and obese children compared to healthy controls. On the contrary, there was no difference in Hsp90*α* levels between overweight and obese children and healthy controls (Table 2).

There were no significant differences between serum Hsp90*α*, Hsp90*β*, and total Hsp90 in obese versus overweight children (Table 3).

No differences were found in serum Hsp90*α* or total Hsp90 levels in patients with insulin resistance and patients without insulin resistance. Patients with insulin resistance tended to have higher Hsp90*β* levels (5.75 ng/mL (0.38-30.73) versus 4.02 ng/mL (0.14-98.37), *p* = 0.09, Mann-Whitney *U* test). Insulin levels were positively correlated with Hsp90*β* (although weak correlation, *r* = 0.24, *p* = 0.045, Spearman rho test).

**Figure 1:**
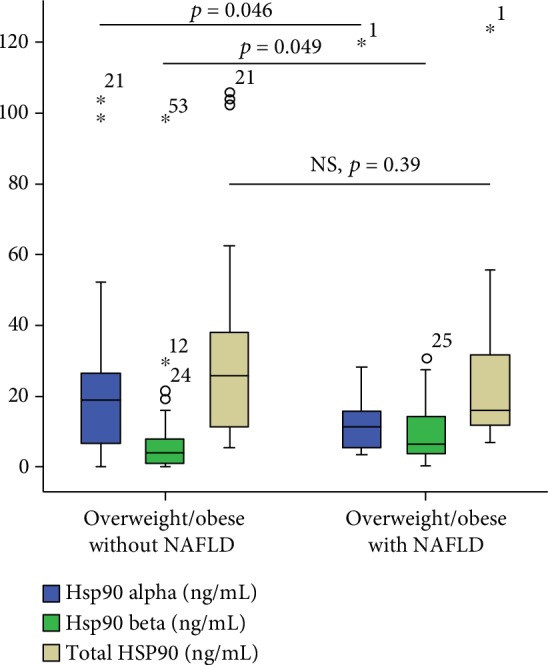
Differences within Hsp90*α*, Hsp90*β*, and total Hsp90 levels in overweight and obese children with or without NAFLD. Numbers in the figure represent outlier values and are patient's identification number from the database.

Significant differences of Hsp90*α* and Hsp90*β* levels between NAFLD and non-NAFLD overweight and obese children patients were found. Higher Hsp90*β* levels were found in NAFLD patients compared to non-NAFLD patients (median 6.46 ng/mL (0.38-30.73) versus 3.95 ng/mL (0.14-98.37), *p* = 0.046, Mann-Whitney *U* test, Figure 1). Interestingly, lower Hsp90*α* levels were found in NAFLD patients compared to non-NAFLD patients (median 11.41 ng/mL (3.59-119.85) versus 18.83 ng/mL (0-105.4), *p* = 0.049, Mann-Whitney *U* test, Figure 1). There were no significant differences between total Hsp90 levels in NAFLD patients compared to non-NAFLD patients (median 16.14 ng/mL (7.06-123.8) versus 25.7 ng/mL (5.52-105.90), Mann-Whitney *U* test, Figure 1).

**Figure 2:**
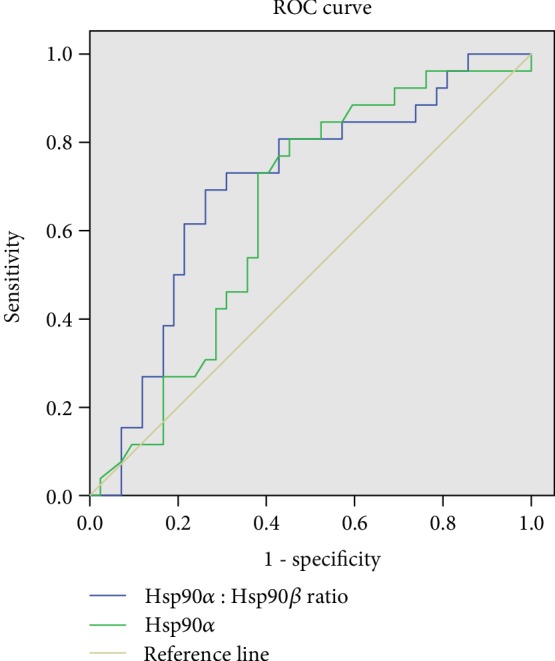
AUC of the Hsp90*α* to Hsp90*β* ratio for NAFLD diagnosis (blue, AUC = 0.70, 95% CI 0.57-0.83) and AUC of Hsp90*α* for NAFLD diagnosis (green, AUC = 0.64, 95% CI 0.55-0.77).

**Figure 3:**
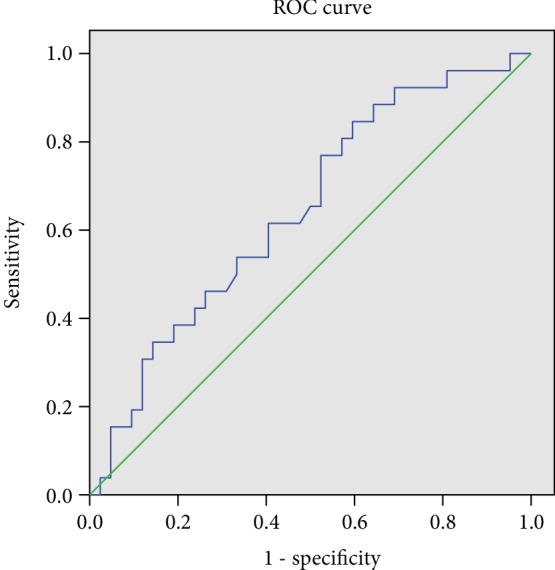
AUC of Hsp90*β* for NAFLD diagnosis (blue, AUC = 0.64, 95% CI 0.51-0.77).

Due to the fact that Hsp90 isoforms had different expression in obese and overweight NAFLD patients (beta isoform was upregulated while alpha isoform was downregulated), it was also decided to calculate the ratio between Hsp90*α* and Hsp90*β* and analyze its diagnostic accuracy for NAFLD compared to the individual biomarkers. Surprisingly, the Hsp90*α* to Hsp90*β* ratio had the best accuracy for NAFLD diagnosis in obese and overweight patients compared to individual biomarkers (Figures 2 and 3). On the contrary, serum levels of Hsp90 had the worst predictive capacity, with an AUC of only 0.56, 95% CI 0.42-0.70 (figure not shown).

## 4. Discussion

Hsp90 had higher expression in overweight and obese children compared to healthy controls. Hsp90*β* levels were higher in these patients, while there were no differences in Hsp90*α* levels compared to healthy controls. Hsp90 isoforms had different expression in NAFLD patients. While Hsp90*β* levels were higher in overweight and obese NAFLD patients, Hsp90*α* levels were lower (Figure 1). Previous studies have shown that Hsp90*β* is the recognized chaperone involved in intracellular signaling and essential cellular processes, whereas Hsp90*α* plays an important role in wound healing [16]. It is also recognized that Hsp90*α* is primarily an extracellular tissue repair enzyme and plays a more reduced role in the cell. This is also explained by the pattern found in children's serum included in the study, with Hsp90*α* levels being higher compared to Hsp90*β* levels in both healthy controls and obese/overweight children.

Hsp90*β* appears to be the Hsp90 isoform involved in insulin resistance as it was proposed as a potential therapeutic target after *in vivo* studies [17]. Inhibition of Hsp90*β* isoform has been shown to improve glucose tolerance and insulin sensitivity in murine diabetes models. Nonetheless, Hsp90*β* has recently been identified as playing a major role in insulin resistance via multiple signal transduction pathways [17]. In this study, serum Hsp90*β* levels were higher in obese and overweight children and also had a trend to be higher in insulin-resistant patients. These results confirm previous *in vivo* reports and indicate that, in overweight and obese children, Hsp90*β* is associated with higher levels of insulin. One possible explanation for the lack of statistical significance is the small sample size (only 24 patients had insulin resistance). Another possible explanation might be the gradual development of insulin resistance and some of the overweight and obese children in the study had not yet developed insulin resistance. Nevertheless, some reports on adults did not find any difference between Hsp90 levels in type 2 diabetes and controls [18]. Future studies on insulin-resistant adult cohorts that will specifically measure Hsp90*β* levels instead of circulating Hsp90 will further answer these questions and clarify the role of Hsp90*β* involvement in insulin resistance.

In the present study, Hsp90 isoforms were confirmed as biomarkers for NAFLD in obese and overweight children. While the Hsp90*β* isoform was higher, the Hsp90*α* isoform was lower in overweight and obese NAFLD patients. Proteomic studies identified Hsp90 isoforms as candidate biomarkers with differential expression in the liver [19, 20]. *In vivo* studies have shown that overexpression of Hsp90 in liver cell lines associated with lipid accumulation while blocking Hsp90 activity determined a decrease of lipogenesis. These *in vivo* findings indicate that Hsp90 plays an important role in the development of NAFLD [21].

As indicated by the predictive values calculated by the AUC from ROC curves for NAFLD diagnosis, it seems that for NAFLD in overweight and obese children the Hsp90*α* to Hsp90*β* ratio can be a more accurate biomarker than Hsp90*α* or Hsp90*β* alone. While the AUC was similar for Hsp90*α* or Hsp90*β*, the predictive capacity improved when considering both biomarkers. The AUC of total Hsp90 was the poorest for NAFLD. All these findings suggest that Hsp90 could be a valuable biomarker when its isoforms are measured. Hepatic lipid accumulation is a chronic process that takes place over several years. It is known that Hsp90*β* isoform is implied in chronic conditions and long-term adaptation responses rather than Hsp90*α* isoform which usually is increased in acute stress condition [22]. It would be interesting to see if this pattern persists in adulthood (these questions would be answered by future studies on adults measuring these specific Hsp90 isoforms).

The current study comprises a series of limitations: firstly, the lack of gold standard diagnosis for NAFLD (liver biopsy). Nonetheless, this study used internationally accepted diagnostic tools (hepatic ultrasound and/or twofold increase of ALAT), tools that are widely tolerated by the patient and its legal guardian and that have showed increased diagnostic capacities. Secondly, the study has a relatively small sample size and only a cross-sectional approach. A prospective approach with two independent sample collections would have been more convincing. However, to the best of our knowledge, this is the first literature study that examined circulating Hsp90 isoforms in obese and overweight children and also the first literature study that confirms Hsp90 isoforms as proteomic biomarkers for NAFLD. Future studies should specifically address the relationship between these two isoforms related to hepatocellular stress and steatogenesis and the prognostic value for NAFLD-related comorbidities of Hsp90*α*/Hsp90*β* isoforms.

In conclusion, in overweight and obese children, Hsp90 isoforms have been confirmed as biomarkers for NAFLD on an independent cohort. It seems more useful to analyze Hsp90 isoforms separately rather than total levels of Hsp90 as the isoforms have a greater discriminative capacity.

## Figures and Tables

**Table 1:** Characteristics of overweight and obese children included in the study.

Characteristic	*N* = 68
Gender (male, %)	35 (51.5%)
Age (years)	10 (3-17)
Weight percentile adjusted for gender and age	97^th^ percentile (87^th^ percentile-99^th^ percentile)
Waist percentile adjusted for gender and age	95^th^ percentile (75^th^ percentile-99^th^ percentile)
Mid arm circumference percentile adjusted for gender and age	95^th^ percentile (25^th^ percentile-99^th^ percentile)
BMI (kg/m^2^)	24.5 (17.60-40.43)
BMI percentile adjusted for gender and age	98^th^ percentile (85^th^ percentile-99^th^ percentile)
BMI *Z*-score	2.03 (1.04-4.00)
WtHR	59.05 (45.5-78.2)
NAFLD (*n*, %)	26 (38.2)
HTA (*n*, %)	32 (47.1%)
A jeune glucose (mg/dL)	88.5 (63-136)
ASAT (UI/L)	23 (13-83)
ALAT (UI/L)	29 (16-224)
ALP (UI/L)	239 (74-470)
Uric acid (mg/dL)	4.8 (2-10.2)
Obese (*n*, %)	59 (86.9%)
Insulin (mU/L)	10.3 (2.05-41.57)
Insulin resistance (*n*, %)	24 (35.3%)
HOMA-IR	2.24 (0.41-13.91)
Hsp90*β* (ng/mL)	4.04 (0.14-98.37)
Hsp90*α* (ng/mL)	14.31 (0-119.85)

BMI: body mass index; WtHR: waist to height ratio; NAFLD: nonalcoholic fatty liver disease; HTA: arterial hypertension; ALP: alkaline phosphatase. Continuous variables are presented as median (minimum-maximum value).

**Table 2:** Serum Hsp90*α*, Hsp90*β*, and total Hsp90 in overweight and obese children compared to healthy controls.

Biomarker	Overweight and obese children	Healthy controls	*p* value (Mann-Whitney *U* test)
Hsp90*α* (ng/mL)	14.31 (0-119.85)	10.71 (5.22-29.10)	0.50
Hsp90*β* (ng/mL)	**4.04 (0.14-98.37)**	**1.78 (0.59-3.09)**	**0.006**
Total Hsp90 (ng/mL)	**22.38 (5.52-123.8)**	**12.28 (7.99-30.99)**	**0.039**

**Table 3:** Serum Hsp90*α*, Hsp90*β*, and total Hsp90 in obese versus overweight children.

Biomarker	Obese children	Overweight children	*p* value (Mann-Whitney *U* test)
Hsp90*α* (ng/mL)	14.09 (0-105.4)	15.98 (4.81-119.85)	0.66
Hsp90*β* (ng/mL)	5.04 (0.14-98.37)	4.02 (0.42-27.46)	0.32
Total Hsp90 (ng/mL)	23.17 (6.36-105.2)	16.4 (5.52-123.87)	0.83

## Data Availability

The data used to support the findings of this study are available from the corresponding author upon reasonable request.
